# Colon cancer cells adopt an invasive phenotype without mesenchymal transition in 3-D but not 2-D culture upon combined stimulation with EGF and crypt growth factors

**DOI:** 10.1186/1471-2407-13-221

**Published:** 2013-05-02

**Authors:** Kirsten Ludwig, Edison S Tse, Jean YJ Wang

**Affiliations:** 1 Moores UCSD Cancer Center, 3855 Health Sciences Drive, La Jolla, CA, 92093-0820, USA; 2 Department of Medicine, Division of Hematology-Oncology, University of California, La Jolla, CA, 92093, USA

## Abstract

**Background:**

The intestinal crypt homeostasis is maintained by a combination of growth factors including Wnt, R-Spondin1, Noggin and the epidermal growth factor (EGF). In human colorectal cancer, the Wnt pathway is constitutively activated through genetic and epigenetic alterations in as many as 11 genes encoding components of this crypt stem-cell maintenance mechanism. Although the proliferation of colon cancer cells does not require Wnt, it is possible that colon cancer cells can still respond to the crypt growth factors in the colonic microenvironment. A number of studies have shown that epithelial cells behave differently in 3-D versus 2-D cultures. Because the 3-D conditions more closely mimic the *in vivo* environment, we examined the effects of Wnt and other crypt growth factors on colon cancer cell growth in 3-D culture.

**Methods:**

Colon cancer cells were grown in 3-D matrigel supplemented with different combinations of crypt growth factors and colonies were examined for morphology and pathways.

**Results:**

When colon cancer cells were cultured in 3-D with EGF, they grew as round spheroid colonies. However, colon cancer cells also grew as flat, disc-like colonies when cultured with EGF plus Wnt, R-Spondin1 and Noggin. Disc colonies were found to have comparable levels of E-cadherin as the spheroid colonies, but showed decreased E-cadherin at the cell-matrix contact sites. Disc colonies also elaborated F-actin rich protrusions (FRP) at the cell-matrix edge, reminiscent of an invasive phenotype but without the expression of vimentin. These E-cadherin and F-actin alterations were not induced by the four growth factors in 2-D culture. Formation of the disc colonies was inhibited by the knockdown of β-catenin and by protein kinase inhibitors such as gefitinib, imatinib and MK-2206. Furthermore, withdrawal of the crypt growth factors was able to revert the disc colonies to spheroid growth, showing that the invasive phenotype was reversible dependent on the availability of growth factors.

**Conclusions:**

These findings show that colon cancer cells remain responsive to the growth factors in the crypt microenvironment and can be induced to undergo morphological transformation in the more physiologically relevant 3-D culture.

## Background

Invasive growth is a critical step in the progression of tumorigenesis as it is what distinguishes a malignant from a benign tumor [1]. A tumor’s ability to disseminate, invade and migrate to distant tissues correlates with worse prognosis [2]. The edge of an invasive tumor is characterized by the loss of apico-basal polarity along with a loss of cell-cell junctions and decreased E-cadherin expression. The actin cytoskeleton is reorganized with the formation of F-actin rich protrusions (FRP) at the leading edge of an invasive tumor, where the cell changes from a cuboidal shape to a motile spindle shape [3]. The cell motility pathways such as those controlled by the integrin receptors, the focal adhesion kinase (FAK), the Rho and Rac family of small G-proteins, and the metalloproteases (MMPs) are also activated in the invasive tumor cells [4]. Histology of colon tumor samples has shown that some of these characteristics, i.e. change in shape and loss of E-cadherin, are found only at the leading edge of the tumor in cells that have direct contact with the ECM, while cells fully encased in the solid tumor maintain expression of E-cadherin [5]. It thus appears that the invasive phenotype might occur in individual cells responding to the external cues rather than the entire tumor mass undergoing global changes.

The recent TCGA (The Cancer Genome Atlas) analysis of human colorectal cancer (CRC) has established that the Wnt and the TGF-β (BMP) pathways are consistently up or down regulated, respectively, by genetic and epigenetic mechanisms in 97% and 87% of CRC in the hypermutated group [6]. The Wnt pathway is also upregulated in 92% of CRC in the non-hypermutated group [6]. This finding is consistent with the fact that maintenance of the intestinal crypt stem cells requires full activation of the Wnt pathway and inactivation of the BMP pathway by the anti-BMP ligand Noggin [7]. In the intestinal crypt compartment, binding of locally produced Wnt and R-Spondin to their respective seven transmembrane-serpetine receptors, Frizzled and Lgr4/5, leads to the assembly of a Wnt signaling complex involving the recruitment of another membrane receptor, LRP, and the stabilization of cytoplasmic β-catenin [8]. The accumulation of cytoplasmic β-catenin is a pre-request for its nuclear translocation, which is regulated by a variety of factors, as β-catenin itself does not contain any nuclear localization signals [9]. Nuclear β-catenin associates with the TCF-family of transcription factors to stimulate gene expression that promotes cell cycle progression and inhibits apoptosis [8]. In the normal regenerating intestinal tissue, Wnt and Noggin levels are high at the base of the crypt to stimulate proliferation and inhibit differentiation. The concentrations of these factors are reduced in the villi, where Wnt and Noggin levels are low and BMP levels are high, promoting differentiation [10]. With the constitutive activation of the Wnt and the receptor tyrosine kinase (RTK) pathways as well as the downregulation of the TGF-β pathway, colon cancer cells do not require this complement of factors to proliferate.

In this study, we show that established colon cancer cells remain responsive to the stimulation of a complement of crypt growth factors to undergo a reversible and localized invasive phenotype but only in 3-D cultures. This invasive response requires activation of β-catenin and EGFR and can be inhibited by drugs that interfere with the function of downstream effectors such as ABL or AKT.

## Methods

### Antibodies and reagents

Anti-β-catenin (610153), and anti-EGFR (610016) were from BD Biosciences. Anti-GAPDH (MAB374), anti-active-β-catenin (05–665), and anti-phospho-FAK (44625G) were from Millipore. Anti-Akt (9272), anti-phospho-Akt (9271), anti-E-cadherin (3195), anti-phospho-Abl (2861), anti-phospho-EGFR (4407), and horseradish peroxidase (HRP)-conjugated secondary antibodies were purchased from Cell Signaling Technology. Anti-FAK (05537) and TRITC conjugated phalloidin (12381) were purchased from Invitrogen. Anti-vimentin (01191) was purchased from GenScript. Anti-Abl 8E9 was generated in our laboratory. The peptides EGF (100–15) and Noggin (250–38) were purchased from Peprotech. Conditioned media was collected from 293 cells stably overexpressing either Wnt3a or R-Spondin1 (a generous gift from Dr. Karl Willert at UCSD) according to [11] using serum free media.

### Cell culture

The human colon cancer cell lines HCT-116 and HT29 (ATCC) were maintained in DMEM medium supplemented with 10% FBS (HyClone). The cell lines LIM1215, 1899, and 2551 were maintained in RPMI 1640 medium (Invitrogen) supplemented with 10% FBS and Additives (10 μM Thioglycerol (Sigma), 2.5 ug/ml Insulin (Sigma) and 0.5 mg Hydrocortisone) [12]. All cell lines were initially maintained in 2-D plastic tissue culture dishes at 37°C with 5% CO_2_. For seeding in 3-D, cells were washed with PBS and trypsonized to detach from each other and the plate. Between 500–1000 cells were seeded in a 24-well plate embedded in 50 μl of 100% matrigel (BD Biosciences). Each well then received 500 μl of DMEM/F12 media supplemented with 1% Pen/Strep (Cellgro), 1M HEPES (Gibco), and Glutamax (Gibco). Growth factors (EGF, Noggin, Wnt3a condition media, and R-Spondin1 condition media) were then individually added to each well. Media was changed every 2 days for a total of 6 days, at which time colonies were passaged.

### Immunofluorescence and confocal microscopy

Cells were grown as described above, fixed with 3% paraformaldehyde for 20 mins. at room temperature and stained according to [13]. Images were captured using an Olympus FV1000 scanning laser confocal microscope.

### Immunoblotting

Proteins from the cell lines were extracted in RIPA buffer (25 mM Tris–HCl pH 7.4, 1 mM EDTA, 0.1% SDS, 150 mM NaCl, 1% NP-40, 1% Sodium Deoxycholate, 1 mM phenylmethylsulfonyl fluoride, and protease inhibitor cocktail) and measured by Lowry protein assay. Equal amounts (50 μg) of total proteins were loaded on SDS-PAGE, transferred onto a nitrocellulose membrane, and probed with primary antibodies overnight at 4°C. HRP conjugated secondary antibodies were incubated for 1 hour at room temperature. Proteins were visualized by chemiluminescence as recommended by the manufacturer (Thermo).

### Luciferase assay

Cells were transfected with β-galactosidase and either BRE (gift from Peter ten Dijke) or TopFlash luciferase plasmid using Genetran (Biomiga) according to manufacturer’s protocol. Twenty four hours later, cells were lysed with Cell Culture Lysis Reagent (Promega) and the luciferase substrate (Promega) was added at a 5:1 dilution. Luminescent values were determined by Monolight 3010 Luminometer. β-galactosidase assay was performed on 96 well plate using ONPG substrate solution (Sigma) and 10μL of cell lysate in each well. Absorbance values were read at 420nm. Luciferase assay was the normalized to β-galactosidase readings.

### Statistical analysis

Data are represented as mean and SEM (Standard Error of the Mean). Two-tailed unpaired t-test was used to determine statistical significance of the differences between data sets. p < 0.05 was considered statistically significant.

## Results and discussion

### Formation of disc-like colonies in 3-D culture

**Figure 1:**
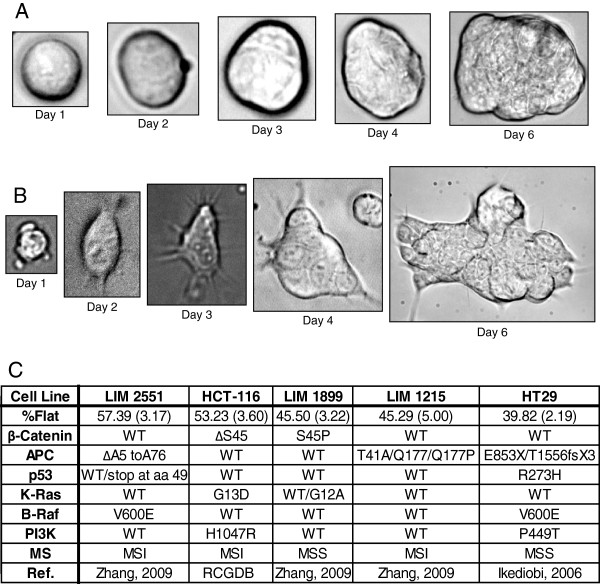
**Established colon cancer cell lines grow as disc (flat) or round (spheroid) colonies in 3D. A**) Phase images of HCT-116 cells grown in 3-D matrigel, with EGF (E), Noggin (N), Wnt3a (W), and R-Spondin1 (R) (RNEW) media. Images were captured on day 1, 2, 3, 4, and 6, and show colonies that grew as round spheres. **B**) Phase images of HCT-116 cells grown in 3-D matrigel as in (**A**). Images show colonies that grew as flat discs. **C**) Summary of colony phenotypes of colon cancer cell lines grown in 3-D matrigel with RNEW media. Top row shows percentage of disc colonies, with SEM in parenthesis, n>3. Subsequent rows depict mutational status of genes known to drive in colon cancer development RCGDB (Roche Cancer Genome Database [16]).

It has recently been demonstrated that mouse intestinal crypt cells can be propagated to form intestinal organoids in 3-D Matrigel culture supplemented with four growth factors; EGF (E), Wnt3a (W), R-spondin1 (R) and Noggin (N) [14]. By contrast, human colon adenocarcinoma cells can be propagated in 3-D Matrigel culture without those four growth factors. This factor-independent growth of human colon cancer cells is consistent with the TCGA data, which showed that the majority of human CRC activate the Wnt and the RTK pathways while inactivating the TGF-β pathway through genetic or epigenetic alterations [6]. To determine if human colon cancer cells remain responsive to the crypt growth factors, we cultured a panel of human colon cancer cell lines (Figure 1C) embedded in 3-D Matrigel in the presence or absence of EGF (E), Wnt3a (W), R-Spondin1 (R) and Noggin (N) (RNEW). Cells grown in the presence of E alone mostly formed round colonies (Figure 1A), similar to the growth phenotype of colon adenocarcinoma cells in 3-D [15]. However, when these established human colon cancer cells were grown in RNEW, we observed the formation of disc-like colonies characterized by a monolayer growth of cells with cytoplasmic protrusions on the edges of the colonies (Figure 1B). These disc colonies were not attached to the bottom of the petri dish because the disc-colony formation was not impeded by coating the petri dish with poly-HEMA to prevent 2-D monolayer growth (Additional file 1: Figure S1). The formation of disc colonies in 3-D RNEW culture was observed with a panel of human colon cancer cell lines containing different mutations in the RTK or the mismatch repair pathways (Figure 1C). These results show that established colon cancer cells remain responsive to the crypt growth factors and that this responsiveness is not affected by the RTK-pathway or the mismatch repair status.

### Formation of disc colonies requires four factors and is reversible

**Figure 2:**
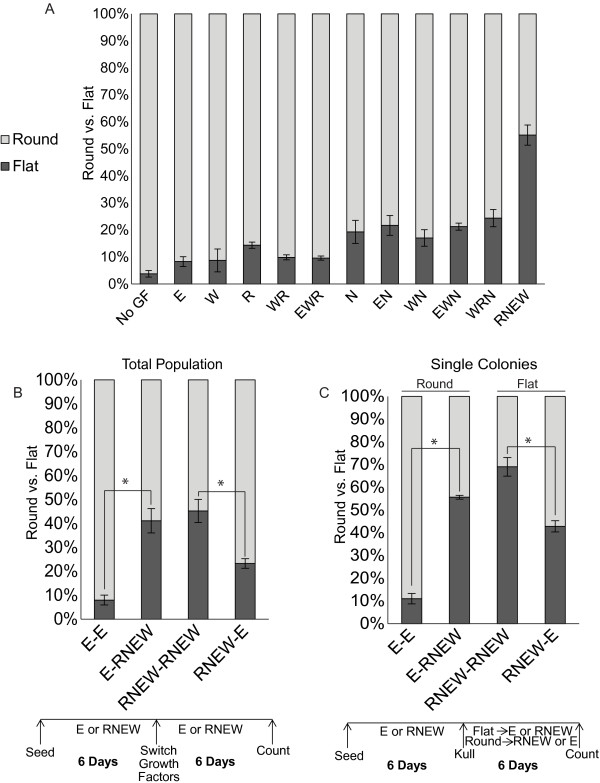
**The disc colony is reversibly induced by EGF plus crypt growth factors. A**) Quantitation of round (spheroid) and flat (disc) colonies of HCT-116 cells grown in 3-D matrigel for 6 days with the indicated combinations of growth factors; R-Spondin1 (R), Noggin (N), EGF (E), and Wnt3a (W). Results shown are mean percent of round or flat colonies +/− standard error of the mean (SEM), n>3. **B**) Quantitation of round and flat colonies of HCT-116 cells in a heterogeneous population grown in E or RNEW media for 6 days and then the reverse media for an additional 6 days as depicted by the scheme below the histogram. Results are expressed as mean percent of round or disc morphology +/− standard error of the mean (SEM), n>3, *p < 0.05. **C**) Quantitation of round or flat colonies of HCT-116 cells from a pure (100%) population of round or disc colonies grown in either E or RNEW media for 6 days as depicted below the histogram. Results are expressed as mean percent of round or disc morphology +/− standard error of the mean (SEM), n>3, *p < 0.05.

Under the 3-D RNEW culture condition, between 40-60% of the colonies took on the disc morphology among the five cell lines tested (Figure 1C). Although EGF alone was not sufficient to induce disc growth, it was nevertheless required for this 3-D growth phenotype (Figure 2A). Individually, each of the four growth factors did not induce a significant level of disc colony formation (Figure 2A). Addition of RNW without E also failed to induce disc growth, as did other combinations of three growth factors (Figure 2A). Only when all four growth factors were present was there a ~50% incidence of disc colonies.

To determine if the ~50% disc growth was due to pre-existing heterogeneity, we conducted two different media-switching experiments as outlined in Figure 2B and 2C. In the first experiment, we cultured cells in E or RNEW media for 6 days, determined the percentage of disc and round colonies and then switched the media and assessed the incidence of disc and round colonies 6 days later (Figure 2B). We found that the occurrence of disc colonies was determined by the growth factors as they reverted back to round colonies after switching from RNEW to E (Figure 2B), showing that the disc morphology was reversible. In the second experiment, we picked individual disc colonies from RNEW and placed them in RNEW or E such that 100% of disc colonies were grown in these media (Figure 2C). In parallel, 100% of round colonies were transferred to RNEW or E media (Figure 2C). These colonies were then grown for an additional 6 days, and the morphology ratio was determined. We found that a fraction of the disc colonies reverted back to round growth when transferred to either RNEW or E media (Figure 2C, disc). Likewise, approximately ~50% of the round colonies became disc when transferred to RNEW media (Figure 2C, round). Together, these results show that switching the growth factors could reverse the growth phenotype. Intriguingly, a pure population of disc colonies grown in RNEW did not all remain disc, as is true for the round colonies. It was never possible to achieve a 100% pure population of disc or round colonies. As the disc to round ratio in RNEW media was consistently around 1 to 1, they are likely to be the result of growth factor-induced epigenetic alterations. However, these results cannot rule out the possibility that the responsiveness to the growth factors is determined by some pre-existing heterogeneity in these established colon cancer cell populations.

### Effects of RNEW and the requirements of oncogenic pathways in 3-D disc growth

**Figure 3:**
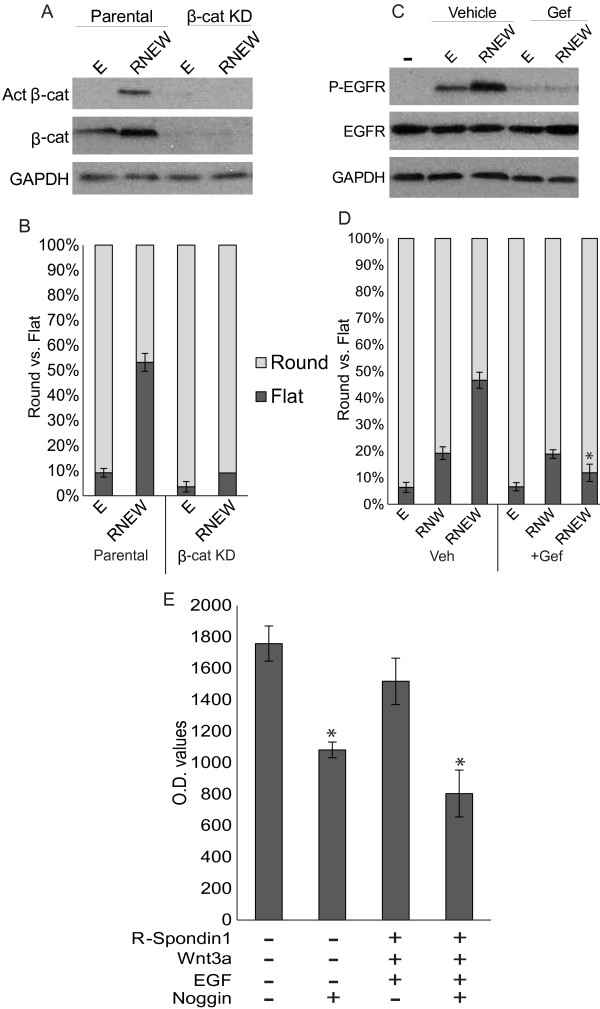
**EGF receptor tyrosine kinase and β-catenin are required for disc colony formation. A**) Western blots of total and activated β-catenin. HCT-116 cells were grown in 3-D matrigel for 6 days in either E or RNEW media. Cells were then lysed and subjected to Western blot analysis to determine the levels of total and activated β-catenin as described in Materials and Methods. GAPDH was used as a loading control. **B**) Quantitation of round or flat colonies of β-catenin knockdown (β-cat KD) HCT-116 cells grown in E or RNEW media 6 days. Results are expressed as mean percent of round or disc morphology +/− standard error of the mean (SEM), n>3, *p < 0.01. **C**) Western blots of total and phospho-EGFR. HCT-116 cells were grown in 3-D matrigel for 6 days in the presence or absence of 50 nM gefitinib. Cells were then lysed and subjected to Western blot analysis to determine the levels of total and phospho-EGFR as described in Materials and Methods. GAPDH was used as a loading control. **D**) Quantitation of round or flat colonies of HCT-116 cells grown in E, RNW or RNEW media in the presence or absence of 50 nM gefitinib for 6 days. Results are expressed as mean percent of round or disc morphology +/− standard error of the mean (SEM), n>3. *=p<.01. **E**) Quantitation of luciferase activity of the BRE-reporter. BRE-luciferase and β-galactosidase reporters were co-transiently transfected into HCT-116 cells. 24 hours later, cells were treated with the indicated growth factors for an additional 24 hours and luciferase and β-galactosidase were measured. Values are normalized to β-galactosidase activity. Results expressed as the mean percent of normalized luciferase activity +/− SEM, n=3.

The knowledge that all four growth factors were required for disc growth raised the question of whether the growth factors were activating their canonical signaling pathways, and if blockage of those pathways could inhibit disc formation. The HCT-116 cells express the Wnt receptor Frizzled [17-19] and the R-Spondin1 receptors Lgr4/5 [20-22]. HCT-116 cells grown in 3-D matrigel for 6 days in the presence of RNEW had a significant increase in the activated and the total β–catenin over cells treated with E alone (Figure 3A). Moreover, a significant reduction in the amount of disc colony formation was found with HCT-116 cells stably knocked-down for β-catenin (Figure 3B), suggesting that β–cat is required for disc growth.

The EGF receptor (EGFR) tyrosine kinase was also activated upon growth in RNEW for 6 days (Figure 3C). When HCT-116 cells were grown in E alone, an increase in phospho-EGFR was observed over no growth factors, however culturing in RNEW increased EGFR activation over growth in E alone, indicating that RNEW could further activate the receptor tyrosine kinase. Furthermore, when cells were grown in the presence of either E or RNEW with 50 nM gefitinib for 6 days, EGFR phosphorylation was abolished, as was the ability to form disc colonies (Figure 3D). To further illustrate the role of EGFR tyrosine kinase in disc colony formation, cells were grown with RNW growth factors in the presence or absence of gefitinib. When stimulated with RNW, we observed a significant decrease in disc colony formation relative to RNEW. Under the RNW condition, gefitinib no longer reduced the number of disc colonies (Figure 3D). These results showed that the EGFR pathway was an important contributor to the formation of disc colonies.

To examine Noggin activity against BMP (bone morphogenetic protein), a BRE (BMP responsive element) driven luciferase assay was conducted to determine if addition of Noggin could decrease BMP activity. We detected BRE-luciferase activity, which was not affected by treatment with REW in HCT-116 cells (Figure 3E). Addition of Noggin either alone or with REW caused a significant reduction in the BRE-luciferase activity (Figure 3E), indicating that Noggin did inhibit BMP activity in this system.

**Figure 4:**
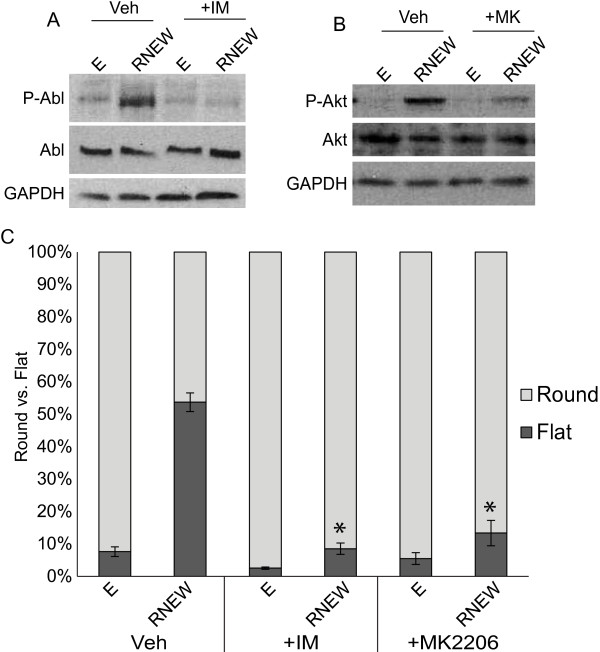
**RNEW activates ABL and AKT which are required for disc colony formation. A**) Western blots of total and phospho-ABL. HCT-116 cells were grown in 3-D matrigel for 6 days in the presence or absence of 1μM imatinib. Cells were then lysed and subjected to Western blot analysis to determine the levels of total and phospho-ABL as described in Materials and Methods. GAPDH was used as a loading control. **B**) Western blots of total and phospho-AKT. HCT-116 cells were grown in 3-D matrigel for 6 days in the presence or absence of 50nM MK-2206. Cells were then lysed and subjected to Western blot analysis to determine the levels of total and phospho-AKT as described in Materials and Methods. GAPDH was used as a loading control. **C**) Quantitation of round or flat colonies of HCT-116 cells grown in E or RNEW media in the presence of vehicle, 1 μM imatinib, and 50 nM MK-2206 for 6 days. Results are expressed as mean percent of round or disc morphology +/− standard error of the mean (SEM), n>3, *p<0.001.

Since the activity of β-catenin and EGFR was required for disc growth, we determined if other oncogenic pathways were activated by RNEW and required for disc formation. To that end, we grew HCT-116 cells in 3-D matrigel for 6 days in the presence of E or RNEW with the ABL inhibitor imatinib and the AKT inhibitor MK-2206. We found that RNEW was able to activate both ABL (Figure 4A) and AKT (Figure 4B), and this activation was abolished when colonies were cultured with the respective inhibitor for 6 days. More importantly, without activation of each pathway, HCT-116 cells were not able to grow as disc colonies (Figure 4C). All together, these data suggest that RNEW activates several oncogenic pathways, including β–catenin, EGFR, ABL, and AKT, can induce cells to grow as disc colonies in a reversible manner, and the activation of each of these pathways is required for disc growth.

### Disc colonies exhibit characteristics of an invasive phenotype when cultured in 3-D but not 2-D

**Figure 5:**
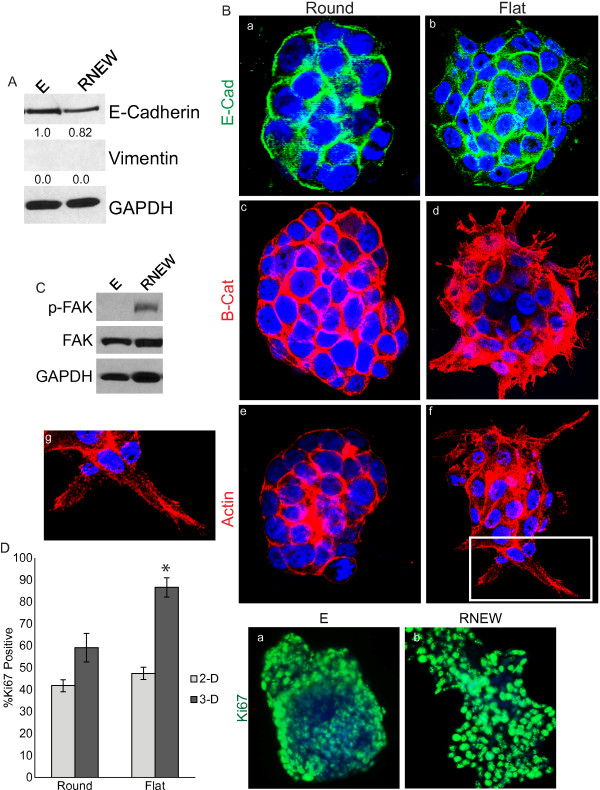
**Invasive characteristics at the edge of the flat disc-like colonies. A**) Western blots of E-cadherin and vimentin. HCT-116 cells were grown in 3-D matrigel with E or RNEW media for 6 days. Cells were then lysed and subjected to Western blot analysis to determine the levels of E-cadherin and vimentin as described in Materials and Methods. GAPDH was used as a loading control. Densitometry shown below each protein. **B**) Confocal images of round or flat colonies of HCT-116 cells grown in 3-D with E or RNEW media for 6 days. a, c, and e: round colonies; b, d, f, and g: flat colonies; a-b: E-cadherin merged with DNA; c-d: β-catenin merged with DNA, and e-g: actin merged with DNA, g: zoom of image f. **C**) Western blots of total and phospho-FAK. HCT-116 cells were grown in 3-D matrigel for 6 days with E or RNEW media. Cells were then lysed and subjected to Western blot analysis to determine the levels of total and phospho-FAK as described in Materials and Methods. GAPDH was used as a loading control. **D**) Quantitation and images of Ki67 positive cells. HCT-116 cells were grown in 2-D or 3-D conditions with E or RNEW media for 6 days, stained for Ki67 and DNA and the percentage of Ki67 positive cells was assessed by immunofluorescence. Results are expressed as mean percentage of Ki67 positive cells, +/− SEM, n=3. Fluorescent images of HCT-116 cells grown in 3-D with (a) E or (b) RNEW media for 6 days. Cells were stained for DNA (blue) and Ki67 (green).

One of the characteristics that distinguished the spheroid versus the disc colonies was the formation of cytoplasmic protrusions, which extended into the matrigel (Figure 1A). This phenotype was reminiscent of invading pseudopodia observed with locally invasive carcinomas that have undergone EMT (epithelial mesenchymal transition) [23]. We therefore examined whether growth in RNEW could affect the expression of the epithelial marker E-cadherin and the mesenchymal marker vimentin [24,25]. Immunoblotting of whole cell lysates revealed a slight reduction of E-cadherin levels and no gain of vimentin expression in 3-D cultures grown with RNEW (Figure 5A). This minimal reduction of E-cadherin was most likely occurring in the cells at the periphery of the colonies, as can be seen in the confocal images. As shown in Figure 5B, E-cadherin expression was lost in cells on the edge of the disc colonies (Figure 5Bb), while spheroid colonies maintained E-cadherin expression throughout the entire colony (Figure 5Ba).

Consistent with a loss of E-cadherin expression, we found that β-catenin was no longer localized to the cell periphery in disc colonies, but instead became more diffusely cytoplasmic and partially nuclear in cells at the leading edge (Figure 5Bd). With the round colonies, which maintained E-cadherin expression, β–catenin remained at the cell periphery (Figure 5Bc). Furthermore, disc colonies displayed actin stress fiber formation (Figure 5Bf-g), while actin was organized as a cortical ring under the plasma membrane of cells in the round colonies. More importantly, cells on the edge of the disc colonies and with actin stress fiber formation took on the shape of a more motile spindle shape with F-actin rich protrusions (FRP) that were reminiscent of highly invasive cells [3]. Actin stress fiber and FRP formation was accompanied with an increase in FAK activation (Figure 5C) in RNEW vs. E stimulated 3-D cultures. Finally, disc colonies displayed a significant increase in Ki67 staining as compared to round colonies (Figure 5D). However this change in proliferation was only observed when cells were grown 3-D but not in 2-D (Figure 5D). Intriguingly, almost all of the cells at the edge of the colonies were Ki67 positive, while only a percentage of the cells on the interior of the colony were positive for the proliferation marker, suggesting a localized invasive transformation.

**Figure 6:**
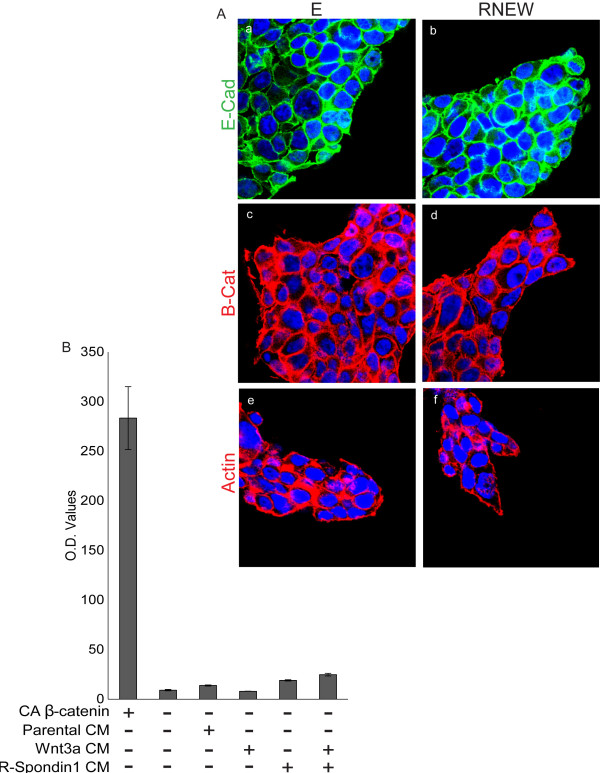
**Growth factors did not induce invasive characteristics in 2-D cultures. A**) Confocal images of HCT-116 cells grown in 2-D with E or RNEW media for 6 days. a, c, and e: cells grown in E media; b, d, and f: cells grown in RNEW media; a-b: E-cadherin merged with DNA; c-d: β-catenin merged with DNA, and e-g: actin merged with DNA. **B**) Quantitation of luciferase activity of the TOPFLASH-reporter. TOPFLASH-luciferase and β-galactosidase reporters were co-transiently transfected into HCT-116 cells. 24 hours later, cells were treated with the indicated growth factors for an additional 24 hours and luciferase and β-galactosidase were measured. Values are normalized to β-galactosidase activity. Results expressed as the mean percent of normalized luciferase activity +/− SEM, n=3.

That RNEW was able to stimulate proliferation in 3-D but not 2-D prompted us to test other effects of RNEW in 2-D culture. We found that the addition of E or RNEW did not affect the cell surface expression of E-cadherin and β-catenin, nor did the growth factors stimulate the formation of actin stress fibers, in 2-D cultures (Figure 6A). We also measured the β–catenin-driven TCF transcription activity using the TOPFLASH-luciferase reporter. Co-expression with a constitutively active β–catenin (S37A) was used as a positive control for activation of the TOPFLASH reporter in HCT-116 cells (Figure 6B). By comparison, stimulation with Wnt3a and R-Spondin1 only led to a minimal activation of the TOPFLASH reporter (Figure 6B) in 2-D cultures. Together these data suggest that RNEW induces the formation of disc colonies, which display characteristics of localized invasion, only when cells are grown in 3-D, which more closely mimics the *in vivo* conditions.

## Conclusions

Our results show that established colon cancer cell lines can be cultured in 3-D matrigel, and like their original source, human CRC, do not require the presence of EGF, R-Spondin1, Wnt3a, or Noggin for proliferation and long term expansion. However these cells did remain responsive to crypt growth factors by taking on a disc-like morphology when grown in the presence of EGF, Wnt, R-spondin1, and Noggin (Figures 1, 2). Chemical or genetic perturbation of the EGFR or the β-catenin pathway revealed that RNEW not only activated these oncoproteins (Figure 3), but that both were required for disc formation. We found that these growth factors also activated ABL and AKT kinases and inhibition of either pathway could prevent disc colony formation (Figure 4). This growth factor-induced disc morphology correlated with localized and reversible invasive characteristics, however it was only seen when cells were grown in 3-D conditions (Figures 5, 6). Given that the amount of disc colony formation with RNEW was an additive but not a synergistic effect indicates that the activation of all three pathways (β-catenin/Wnt, EGFR, and BMP) may function independently, as opposed to converging on a common target.

Interestingly, RNEW was not able to increase proliferation or stimulate actin re-organization when HCT-116 cells were grown in 2-D. This observation is consistent with breast cell lines like MCF10A, which, when grown in 2-D conditions remain as unpolarized epithelial cells; however under 3-D conditions form acini and undergo differentiation and polarization [13]. Additional file 2: Figure S2 shows that when HCT-116 cells are plated on matrigel coated coverslips RNEW is still not able to induce an invasive phenotype. This suggests that matrigel per se is not the collaborating factor for RNEW to induce the cytoskeletal transformation. An obvious difference between 2-D and 3-D tissue culture conditions is the stiffness of the culture substratum. The stiffness of healthy intestinal tissue is between 20–40 kPa [26,27], while standard plastic tissue culture dishes are around 10,000 kPa [28]. Another difference between 2-D and 3-D growth conditions is the area of cell contact with the ECM. When cells were grown on either plastic or matrigel coated slides only a fraction of the cell membrane was in direct contact with the matrix. However, in 3-D a significantly larger portion of the cell surface was engaged in ECM interactions. Increased cell surface contact with the ECM might alter the signaling activation of growth factors receptors as has been reported [29], and stimulate invasion of cells on the edges of the colonies.

Colon cancer presents as two different morphologies; the of stalked (pedunculated) type, and the flattened disc type [31]. While the majority of colon cancers are of the pedunculated morphology which are easy to detect by colonoscopy [30], between 10-20% of colon cancers are of the disc type [31,32]. This form of cancer is very difficult to detect, corresponds with a worse prognosis [32,33], and has been postulated to be up to 10 times more malignant than the pedunculated type [33]. The genetics for these tumor sub-types are not yet completely understood as some groups have found that disc tumors have increased nuclear β-catenin and no KRAS or BRAF mutations [32], while other groups have found the opposite [33]. What is known is that these tumors remain disc throughout the entirety of the tumorigenesis process and do not follow the traditional Vogelstein model of colon carcinogenesis [32]. Our finding suggests that the tumor phenotypes can be influenced not only by the genetic mutations but also by the availability and the tumor response to crypt growth factors. Using the method of 3-D culture with RNEW developed in this study, we were able to identify a previously unreported role for imatinib and MK-2206 in inhibition of growth factor-induced invasive phenotype. This suggests that this methodology may be used to study the effects of other inducers and/or inhibitors of tumor invasion.

## Abbreviations

EGF: Epidermal Growth Factor; MMP: Matrix-Metallo Proteases; FAK: Focal Adhesion Kinase; FRP: F-actin Rich Protrusions; ECM: Extra Cellular Matrix; TCGA: The Cancer Genome Atlas; CRC: Colo-Rectal Cancer; TGF-β: Transforming Growth Factor; BMP: Bone Morphogenetic Protein; RTK: Receptor Tyrosine Kinase; R: R-Spondin1; N: Noggin; E: EGF; W: Wnt3a; RNEW: R-Spondin1, Noggin, EGF, Wnt3a.

## Competing interests

The authors declare no conflicts of interest.

## Author contributions

KL carried out the studies, and wrote the manuscript, EST carried out the luciferase assays, and JYJW conceived of the study, participated in its design, and revised the manuscript. All authors read and approved the final manuscript.

## Pre-publication history

The pre-publication history for this paper can be accessed here:

http://www.biomedcentral.com/1471-2407/13/221/prepub

## Supplementary Material

Additional file 1: Figure S1 Disc colonies are not a result of cells sitting on the bottom of the well. **A**) Phase images of HCT-116 cells grown in 3-D matrigel on different surfaces; a: non-coated plastic; b: non-coated plastic, cells sitting on the bottom of the plate; c: polyHEMA coated plastic; d: matrigel coated plastic; e: glass cover slip with RNEW media. Click here for file

Additional file 2: Figure S2 Growth on 2-D matrigel coated slides does not induce invasive characteristics. **A**) Confocal images of round or disc colonies of HCT-116 cells grown on slides coated with matrigel in E or RNEW media for 6 days. a, c, and e: cells grown in E media; b, d, and f: cells grown in RNEW media; a-b: E-cadherin merged with DNA; c-d: β-catenin merged with DNA, and e-g: actin merged with DNA. Click here for file
