# Supplementary material for: Colon cancer cells adopt an invasive phenotype without mesenchymal transition in 3-D but not 2-D culture upon combined stimulation with EGF and crypt growth factors
Source: BMC Cancer. 2013 May 2;13:221. doi: 10.1186/1471-2407-13-221 (PMC3667045; doi:10.1186/1471-2407-13-221)
Supplement: Additional file 1: Figure S1 — Disc colonies are not a result of cells sitting on the bottom of the well. A) Phase images of HCT-116 cells grown in 3-D matrigel on different surfaces; a: non-coated plastic; b: non-coated plastic, cells sitting on the bottom of the plate; c: polyHEMA coated plastic; d: matrigel coated plastic; e: glass cover slip with RNEW media. [file 1471-2407-13-221-S1.pdf]

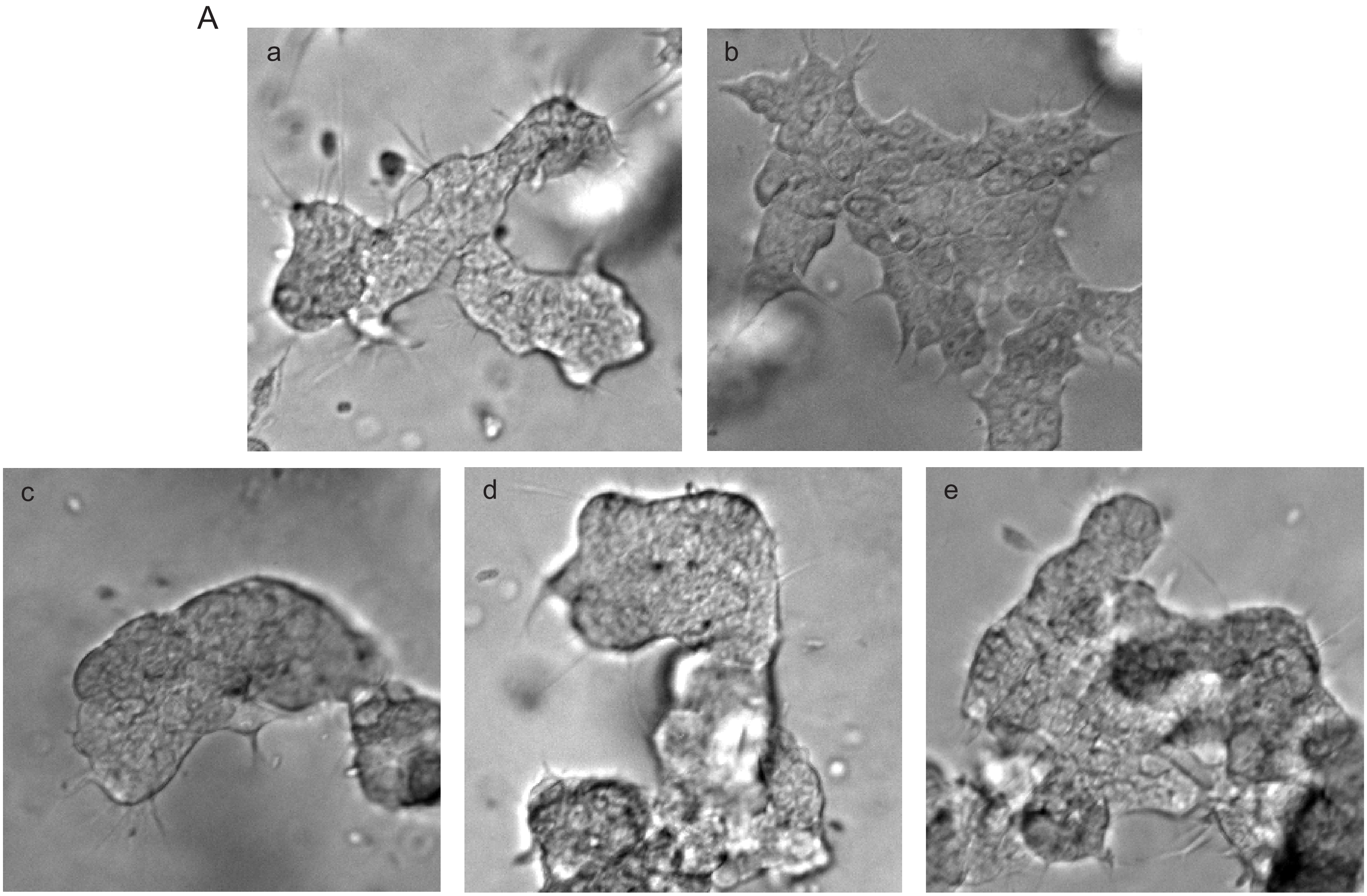

Supplemental Figure 1: Disc colonies are not a result of cells sitting on the bottom of the well.
A) Phase images of HCT-116 cells grown in 3-D matrigel on different surfaces; a: non-coated plastic; b: non-coated plastic, cells sitting on the bottom of the plate; c: polyHEMA coated plastic; d: matrigel coated plastic; e: glass cover slip with RNEW media.
