# Supplementary material for: Colon cancer cells adopt an invasive phenotype without mesenchymal transition in 3-D but not 2-D culture upon combined stimulation with EGF and crypt growth factors
Source: BMC Cancer. 2013 May 2;13:221. doi: 10.1186/1471-2407-13-221 (PMC3667045; doi:10.1186/1471-2407-13-221)
Supplement: Additional file 2: Figure S2 — Growth on 2-D matrigel coated slides does not induce invasive characteristics. A) Confocal images of round or disc colonies of HCT-116 cells grown on slides coated with matrigel in E or RNEW media for 6 days. a, c, and e: cells grown in E media; b, d, and f: cells grown in RNEW media; a-b: E-cadherin merged with DNA; c-d: β-catenin merged with DNA, and e-g: actin merged with DNA. [file 1471-2407-13-221-S2.pdf]

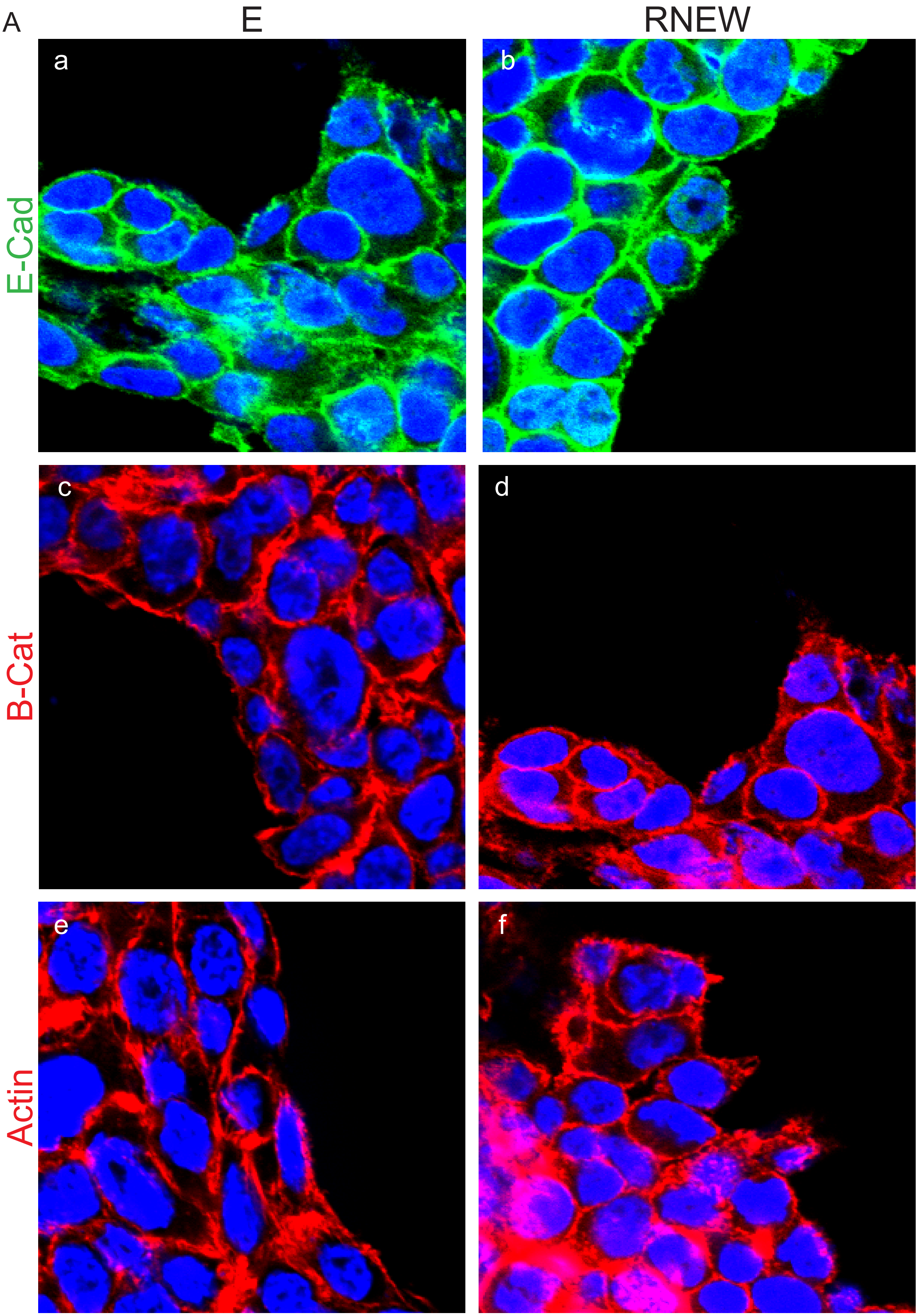


**Supplemental Figure 2: Growth on 2-D matrigel coated slides does not induce invasive characteristics.** A) Confocal images of round or disc colonies of HCT-116 cells grown on slides coated with matrigel in E or RNEW media for 6 days. a, c, and e: cells grown in E media; b, d, and f: cells grown in RNEW media; a-b: E-cadherin merged with DNA; c-d: β-catenin merged with DNA, and e-g: actin merged with DNA.
